# Dasinya: A dataset and preprocessing pipeline for the Badini Kurdish dialect

**DOI:** 10.1016/j.dib.2026.113129

**Published:** 2026-08-03

**Authors:** Vaman A. Saeed, Karwan Jacksi

**Affiliations:** a Information Technology Department, Technical College of Duhok, Duhok Polytechnic University, Duhok, Kurdistan Region, Iraq; b Semantic Web Lab, University of Zakho, Kurdistan Region, Iraq

**Keywords:** Badini Kurdish, Corpus, NLP preprocessing, Sentence segmentation, Tokenisation, Low-resource language, Perso-Arabic script, Kurdish language technology

## Abstract

Despite notable advances in NLP for low-resource languages, dialects written in non-Latin scripts — especially those without standardised digital resources — continue to receive insufficient attention. The Badini Kurdish dialect, written in Perso-Arabic script and spoken across the Kurdistan Region of Iraq, lacks any dedicated NLP preprocessing pipeline in the literature. This paper presents Dasinya, the first openly released Badini Kurdish text corpus, together with the Badini Dialect Processing Toolkit (BDPT) — the first NLP preprocessing pipeline built specifically for this dialect. Dasinya contains 107 files, 87,545 sentences, and roughly 1.28 million words, spanning two source types and nine sub-genres: six book sub-genres (literary, historical, cultural, scientific, psychological, and educational) and three non-book sub-genres (news, academic/linguistic, and broadcast). These files were drawn from two repositories: the Badirxanian Public Library in Duhok (98 books) and the Semantic Web Lab at the University of Zakho (4 books and 5 non-book files). BDPT is structured as a six-stage pipeline that handles validation, Unicode normalisation — enforcing Badini-specific character mappings and preserving the Zero-Width Non-Joiner as a morpheme boundary marker — structural cleaning, deep cleaning, text preprocessing, and sentence segmentation. Orthographic problems particular to Badini Kurdish are addressed at each stage; none of these are handled by existing Arabic, Persian, or Sorani tools. At Stage 6, sentence segmentation reaches a specialist-validated Acc% of 91.4% across all 107 files, measured under a two-metric framework (OK% and Acc%) with genre-sensitive thresholds that reflect genuine linguistic differences between books, news, broadcast, and academic texts. Both the Dasinya corpus and the BDPT pipeline are openly available at https://doi.org/10.5281/zenodo.20729060 and are intended to underpin future Badini Kurdish NLP work, including tokenisation, part-of-speech tagging, stemming, and stopword removal.

Specifications Table

**Table:** 

Subject	Computer Sciences
Specific subject area	Natural Language Processing; Computational Linguistics
Type of data	.docx / .doc — source book and document files (legacy Word format) .txt — plain-text pipeline output per stage (UTF-8, Perso-Arabic script) .txt (structured) — Stage 6 output tagged with <T>/<P>/<S> boundary markers Table — per-file and per-genre corpus statistics
Data collection	Source texts were collected from two institutional repositories: (1) Badirxanian Public Library, Duhok — 98 books and documents in .doc/.docx format spanning six book sub-genres (literary, historical, cultural, scientific, psychological, and educational); (2) Semantic Web Lab, University of Zakho — 9 digitised documents: 4 books, which fall within the six book sub-genres above, and 5 non-book files spanning three non-book sub-genres (news, academic/linguistic, and broadcast). All materials are in the Badini Kurdish dialect written in Perso-Arabic script. No data were collected from individuals; all sources are institutional or published works.
Data source location	Badirxanian Public Library (government public library), Duhok, Kurdistan Region, Iraq. Semantic Web Lab (SWLab), University of Zakho, Kurdistan Region, Iraq.
Data accessibility	Repository name: Dasinya — Badini Kurdish Corpus and BDPT Pipeline (Zenodo) Data identification number: 10.5281/zenodo.20,729,060 Direct URL to data: https://doi.org/10.5281/zenodo.20729060
Software / dependencies	Python 3.14. Pipeline stages implemented using the Python standard library (re, unicodedata, json, csv, glob, os, shutil, argparse, pathlib, collections, datetime). Direct third-party libraries: python-docx 1.2.0 (Word-format ingestion and document output), openpyxl 3.1.5 (spreadsheet logging), and pywin32 312 (Windows COM automation, used for the legacy .doc to .docx conversion). All pipeline code is available in the Zenodo repository (DOI: 10.5281/zenodo.20,729,060). The legacy .doc-to-.docx conversion requires Microsoft Word running on Windows (via pywin32); all subsequent pipeline stages (Stages 1–6) are platform-independent and depend only on Python and the modules listed above.
Related research article	None

## 1 Value of the Data

• Dasinya is the first openly released Badini Kurdish text corpus and the first to be paired with a complete, publicly released NLP preprocessing pipeline. BDPT is the first dedicated preprocessing infrastructure for the Badini dialect, addressing a critical gap in Kurdish language technology for a low-resource, Perso-Arabic script dialect with no prior computational tools.
• The corpus and all pipeline outputs (validated, cleaned, segmented, and tokenised text) can be directly used by other researchers to train and evaluate NLP models for Badini Kurdish — including part-of-speech taggers, named entity recognisers, and language models — and serve as a reproducible benchmark for comparing future preprocessing approaches.
• BDPT’s six documented, reproducible pipeline stages — each evaluated quantitatively — provide a reusable methodological framework adaptable for other low-resource dialects written in Perso-Arabic script, such as related Kurmanji varieties or other minority languages of the region.
• Stage 6 sentence segmentation achieves a specialist-validated accuracy of 91.4% (Acc%) across all 107 corpus files, providing a reusable evaluation methodology for future Kurdish sentence boundary detection research.
• Both Dasinya and BDPT are publicly released on Zenodo, enabling the research community to reproduce all pipeline results and build downstream NLP tools including a POS tagger, stemmer, and stopword list for Badini Kurdish.

## 2 Background

With an estimated 20 to 30 million speakers spread across Iraq, Iran, Turkey, and Syria, Kurdish is one of the most widely spoken low-resource languages in the world, yet its computational infrastructure remains sparse. The dominant written dialects are Sorani (Central Kurdish, Perso-Arabic script) and Badini/Kurmanji (Northern Kurdish, written in Perso-Arabic script in Iraq). Sorani has attracted a growing body of NLP work — KLPT [1] offers normalisation and tokenisation, while DASTAN [2] is the first POS-annotated Sorani corpus, and a comprehensive 97-tag POS tagset for Central Kurdish has recently been proposed [3] — whereas the Badini dialect has almost no dedicated computational resources. Table 1 surveys existing Kurdish NLP corpora and preprocessing tools.

**Table 1:** Comparison of Kurdish NLP corpora and preprocessing tools.

Resource	Dialect	Script	Size	Preprocessing / tools	Open	Year
Pewan corpus [4]	Sorani + Kurmanji	Both	∼3 M words	Raw text only	Yes	2013
KTST [5]	Sorani	Perso-Arabic	∼3.1 K docs	IR test collection; no NLP pipeline	Partial	2014
AsoSoft corpus [6]	Sorani	Perso-Arabic	188 M tokens	Unicode normalisation only	Yes	2020
KLPT [1]	Sorani	Perso-Arabic	Lexicon + rule sets	Tokenise, normalise, stem (no POS module)	Yes	2020
UOZBDN [7]	Badini	Perso-Arabic	921 sentences 51,761 words 53,052 tokens 231 articles, 5 domains	KLPT used internally for normalisation, tokenisation, sentence split; no released Badini pipeline	Yes	2024
DASTAN [2]	Sorani	Perso-Arabic	74,258 words 218 articles 3,163 sentences 38 tags	POS-annotated corpus; hybrid HMM + rule-based tagger (96% accuracy)	Yes	2023
KurCorpus 2B [8]	Sorani + Badini + Hawrami	Perso-Arabic	>2B tokens (per-dialect split not reported)	Generic Unicode normalisation & orthographic correction; no Badini-specific pipeline	Yes	2025
KSTRV1 [9]	Badini + Sorani	Perso-Arabic	1420 images; 19,872 word samples; 20,000 synthetic instances	Scene text recognition; no text pipeline	Yes	2025
**Dasinya + BDPT (this work)**	Badini	Perso-Arabic	87,545 sentences ∼1.28 M words 107 files, 2 source types, 9 sub-genres	Full 6-stage pipeline: validation, Unicode, structural & deep clean, preprocessing, segmentation (91.4% Acc)	Yes	2026

As shown in Table 1, the majority of Kurdish NLP resources have concentrated on Sorani, offering raw text or partial preprocessing at best. Table 1 is ordered chronologically. Dasinya + BDPT is the only resource that pairs a Badini Kurdish text corpus with a fully documented, publicly released multi-stage preprocessing pipeline*.* KLPT [1] provides tokenisation and normalisation but is built for Sorani and cannot be transferred to Badini, where orthographic conventions and Unicode codepoints differ. An earlier Badini corpus, UOZBDN [7], comprises 921 sentences (51,761 words) but was described only in a publication and never released as an openly available dataset, nor accompanied by any preprocessing code. Dasinya is therefore the first Badini Kurdish corpus made openly available to the research community. KurCorpus 2B [8], while the largest Kurdish corpus in existence at over two billion tokens, covers three dialects without Badini-specific processing and reports no per-dialect statistics.

Kurdish NLP has expanded considerably in 2025–2026, with new resources appearing across tasks ranging from named entity recognition and stance detection to semantic similarity, speech translation, and large-scale language modelling. Table 2 brings together six of the most significant recent releases. Taken together, these developments signal a rapidly maturing research ecosystem — but they also expose a stubborn blind spot: nearly every resource either targets Sorani or broad Kurmanji, and none addresses Badini-specific text processing. This is not a historical oversight that has since been corrected; it remains an active gap at the time of writing.

**Table 2:** Recent Kurdish NLP resources (2025–2026).

Dataset	Year	Dialect(s)	Size	NLP Task	Published
KurCorpus 2B [8]	2025	Sorani, Badini, Hawrami	>2B tokens	Language modeling; pretraining (KurBERT)	Mendeley Data doi:10.17632/fb5xhhn6m5.1
Adyan [10]	2025	Sorani	654,404 tokens 2,300+ articles 15 entity types	Named Entity Recognition (NER)	Data in Brief (Elsevier)
Bochun [11]	2025	Sorani	2174 articles (political & economic)	Stance detection	Data in Brief (Elsevier)
KurdSTS [12]	2025	Sorani (Central Kurdish)	10,000 sentence pairs	Semantic textual similarity (STS)	arXiv2510.02,336
AgaCKNER [13]	2025	Sorani (Central Kurdish)	64,563 tokens 2,534 sentences	Named Entity Recognition (NER)	arXiv2511.22,315
FLEURS-Kobani [14]	2026	Kurmanji (Northern Kurdish)	5162 utterances 18+ hours 31 speakers	ASR & speech translation	DialRes-LREC 2026

As is clear from Table 2, resources span multiple dialects, modalities, and NLP tasks, reflecting rapid growth in Kurdish language technology. Note that no resource in this table provides a Badini-specific text preprocessing pipeline.

Dasinya and BDPT were created to fill exactly the gap that both tables expose. The corpus draws on 98 books and documents from the Badirxanian Public Library and 9 files from the Semantic Web Lab at the University of Zakho, covering two source types and nine sub-genres to ensure genre diversity. BDPT is the first preprocessing pipeline for Badini Kurdish built from the ground up to be fully reproducible, running six documented stages from Unicode normalisation through sentence segmentation. A key design requirement at every stage is the preservation of the Zero-Width Non-Joiner (ZWNJ, U + 200C), which serves as a morpheme boundary marker in Badini Kurdish — a writing convention established by Bedirxan [15] — setting BDPT apart from tools designed for Arabic, Persian, or Sorani.

## 3 Data Description

**Fig. 1:**
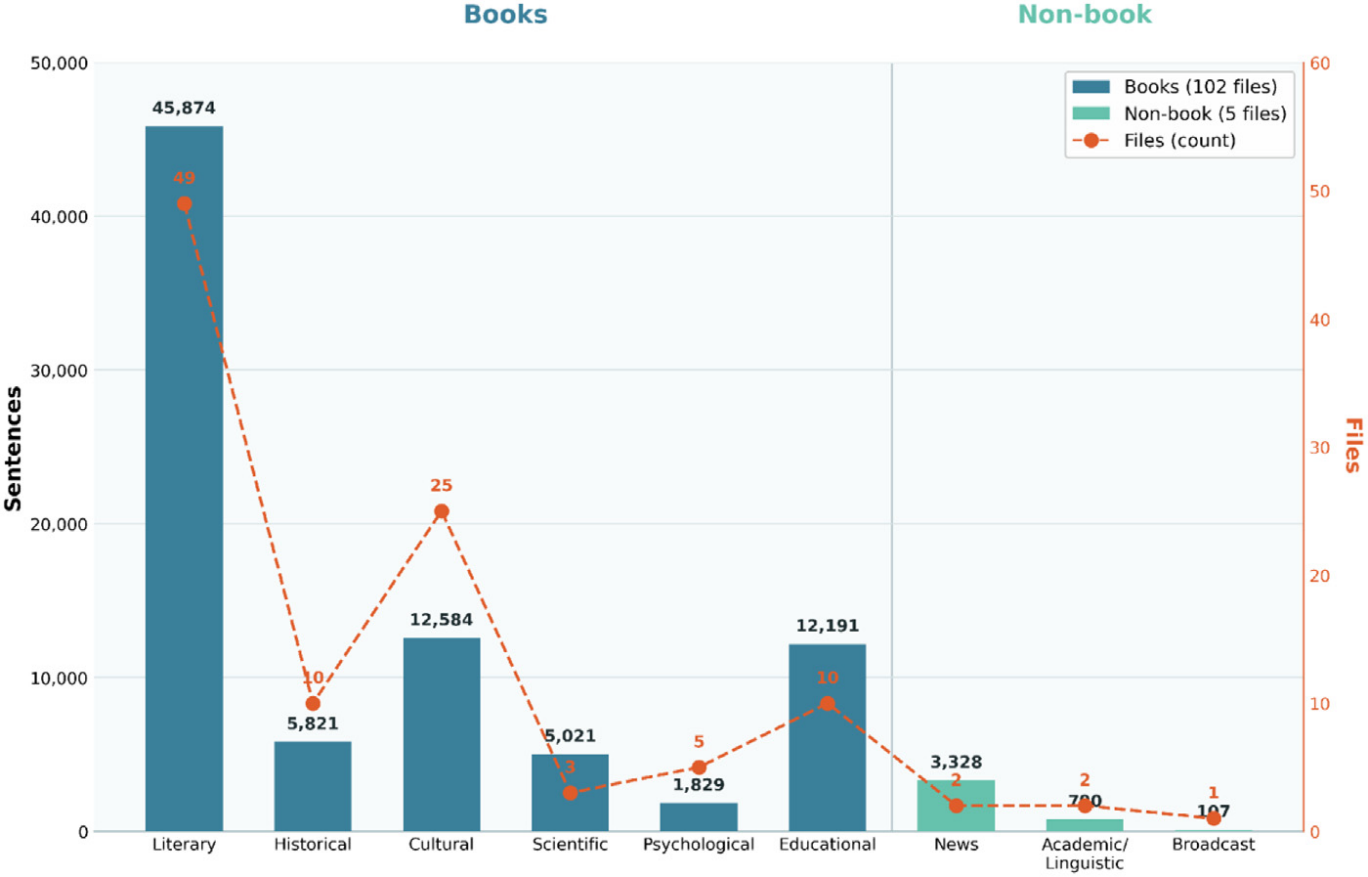
Distribution of sentences and files across the two source types and nine sub-genres of the Dasinya corpus.

Fig. 1 shows the distribution of sentences and files across the two source types and nine sub-genres of the Dasinya corpus. Books constitute the largest source type — 83,320 sentences across 102 files under the content-based grouping used for the segmentation evaluation in Table 8 and Fig. 4. This content-based split (102 Books, 5 Non-book) is deliberately distinct from the institutional source split reported elsewhere in the article — 98 files from the Badirxanian Public Library and 9 from the Semantic Web Lab, UOZ. Both partition the same 107 files and 87,545 sentences; because content type is assigned independently of institutional origin, the two file counts do not coincide. More precisely, the Dasinya corpus comprises 107 source files totaling 87,545 sentences and approximately 1.28 million words. Files span two source types and nine sub-genres. Table 3 presents the distribution of sentences across these sub-genres.

**Table 3:** Genre and sub-genre distribution of the Dasinya corpus across its two source types and nine sub-genres.

Genre	Source type	Sentences	Share (%)
Literary	Books	45,874	52.4%
Historical	Books	5821	6.6%
Cultural	Books	12,584	14.4%
Scientific	Books	5021	5.7%
Psychological	Books	1829	2.1%
Educational	Books	12,191	13.9%
News	Non-book	3328	3.8%
Academic/Linguistic	Non-book	790	0.9%
Broadcast	Non-book	107	0.1%
**TOTAL**		**87,545**	**100%**

The Dasinya repository is organised into two top-level directories: corpus/ (raw and processed text files produced by each pipeline stage) and bdpt/ (the BDPT pipeline source code). The corpus/ directory contains the following subdirectories, one per pipeline stage: raw/, validated/, stage2_unicode/, stage3_clean/, stage4/, stage5_preprocessed/, and structured/ (Stage 6 segmentation output). The bdpt/ directory contains the pipeline modules (stage1_validate.py through stage6_segment.py), configuration files, and the Badini lexicon and morpheme resources.

## 4 Experimental Design, Materials and Methods

### 4.1 Kurdish language, dialects, and orthographic characteristics

Kurdish is a Northwestern Iranian language of the Indo-European family, with an estimated speaker population of 20 to 30 million distributed across Iraq, Iran, Turkey, Syria, and diaspora communities worldwide [7]. No single unified writing system exists for the language. Linguists recognise four principal dialect groups: Kurmanji (Northern Kurdish), Sorani (Central Kurdish), Southern Kurdish, and Hawrami (Gorani). Sorani is written exclusively in Perso-Arabic script, while Kurmanji adopts a Latin-based orthography in Turkey and Syria but shifts to Perso-Arabic in Iraq — the local form known as Badini, centred on the Duhok region. Because these dialects diverge substantially in phonology, morphology, and vocabulary, an NLP tool built for one cannot simply be reused for another.

Badini is the principal Kurdish variety in Duhok Province of the Kurdistan Region of Iraq and in the Hakkari district of southeastern Turkey. Written in Perso-Arabic script, it is orthographically distinct from both Latin-script Kurmanji and Sorani. Its grammar is characterised by agglutinative morphology, a split-ergative case system, and rich verb morphology in which numerous prefixes and suffixes attach directly to verb stems — a combination that creates tokenisation challenges not found in Arabic or Persian. A further complication is that no universally agreed written standard exists; spelling varies across sources, and a large proportion of legacy texts were typed using keyboard layouts that encode Badini-specific characters through Arabic Unicode codepoints that are visually similar but functionally incorrect.

**Fig. 2:**
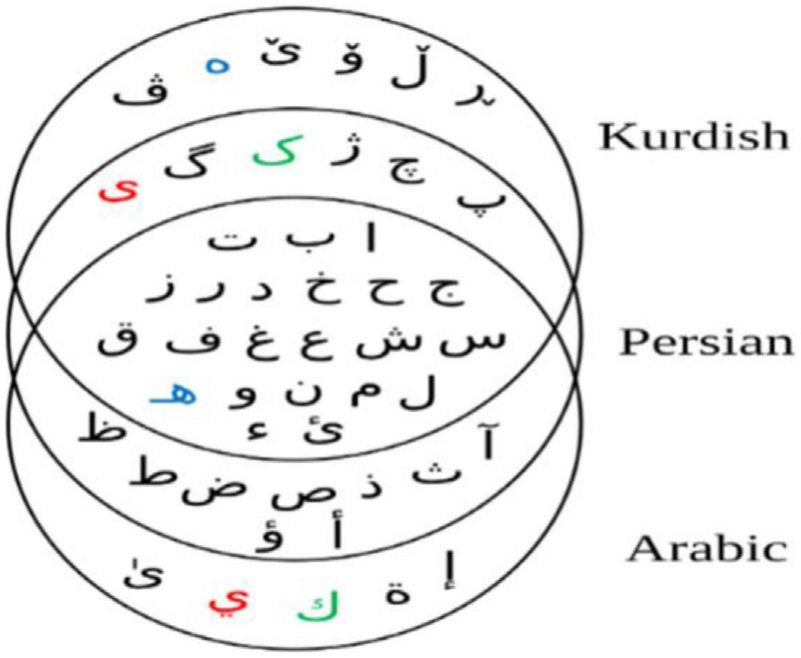
Shared and language-specific characters across Arabic, Persian, and Badini Kurdish.

As illustrated in Fig. 2, the Badini Kurdish alphabet shares a large portion of its characters with Arabic and Persian. However, Badini requires eight additional characters and conventions absent from standard Arabic keyboards: ڕ (heavy R, U + 0695), ڤ (V, U + 06A4), ڵ (heavy L, U + 06B5), ۆ (Kurdish O, U + 06C6), ە (Kurdish Ae, U + 06D5), ی (Kurdish Yeh, U + 06CC), ێ (Kurdish Ê, U + 068E), and the Zero-Width Non-Joiner (ZWNJ, U + 200C) used as a morpheme boundary marker — a convention established for written Kurdish by Bedirxan [15]. Legacy Kurdish keyboards frequently substitute these with visually similar Arabic characters, introducing codepoint-level errors that are invisible to human readers but disruptive to any NLP processing pipeline. The complete Badini alphabet is documented in Table 4, and the eight characters requiring normalisation are listed in Table 5.

**Table 4:** The Badini Kurdish alphabet in Perso-Arabic script. Amber-highlighted rows indicate characters that require Kurdish-specific Unicode codepoints absent from standard Arabic keyboards. These are the characters corrected by BDPT Stage 2.

Letter	Unicode	Kurdish name	Transliteration	Arabic keyboard?
ئ	U + 0626	Hamza	'	Yes
ا	U + 0627	Elif	a	Yes
ب	U + 0628	Bê	b	Yes
پ	U + 067E	Pê	p	No — Kurdish only
ت	U + 062A	Tê	T	Yes
ج	U + 062C	Cim	c / j	Yes
چ	U + 0686	Çê	ch / ç	No — Kurdish only
ح	U + 062D	Hê	h	Yes
خ	U + 062E	Xê	x	Yes
د	U + 062F	Dal	d	Yes
ر	U + 0631	Rê	r	Yes
**ڕ**	U + 0695	Rr (heavy R)	r̄	No — Kurdish-specific
ز	U + 0632	Zê	z	Yes
ژ	U + 0698	Jê	j / zh	No — Kurdish only
س	U + 0633	Sin	s	Yes
ش	U + 0634	Şin	sh / ş	Yes
ع	U + 0639	Eyn	'e	Yes
غ	U + 063A	Xeyn	gh	Yes
ف	U + 0641	Fê	f	Yes
**ڤ**	U + 06A4	Vê	v	No — Kurdish-specific
ق	U + 0642	Qaf	q	Yes
ک	U + 06A9	Kaf (Kurdish)	k	Yes
گ	U + 06AF	Gaf	g	No — Kurdish only
ل	U + 0644	Lam	l	Yes
**ڵ**	U + 06B5	Ll (heavy L)	ł	No — Kurdish-specific
م	U + 0645	Mim	m	Yes
ن	U + 0646	Nun	n	Yes
و	U + 0648	Waw	w/o/û	Yes
**ۆ**	U + 06C6	Oe (Kurdish O)	O	No — Kurdish-specific
ه	U + 0647	Hê	h/e	Yes
**ە**	U + 06D5	Ae (Kurdish E)	e/a	No — Kurdish-specific
**ی**	U + 06CC	Yê (Kurdish)	y/î	No — Kurdish-specific
**ێ**	U + 068E	Ê (Kurdish Ê)	ê	No — Kurdish-specific

**Table 5:** Kurdish-specific characters and the ZWNJ morpheme boundary marker. All eight require codepoint correction by BDPT Stage 2. Rows show the correct Unicode codepoint, the common incorrect form produced by legacy keyboards, and the corresponding BDPT pipeline rule.

Char	Unicode	Description	Common incorrect form	BDPT correction
ڕ	U + 0695	Heavy R (Rr)	U+0631ر	Stage 2 Rule 8
ڤ	U + 06A4	Vê	U+0641ف	Stage 2 Rule 6
ڵ	U + 06B5	Heavy L (Ll)	U+0644ل	Stage 2 Rule 3
ۆ	U + 06C6	Kurdish O	U+0648و	Stage 2 Rule 7
ە	U + 06D5	Kurdish Ae	U+0647ه	Stage 2 Rule 5
ی	U + 06CC	Kurdish Yeh	U+064Aي	Stage 2 Rule 14
ێ	U + 068E	Kurdish Ê	U+0649ى	Stage 2 Rule 1
	U + 200C	ZWNJ (morpheme boundary marker)	'U+0027 or absent	Stage 2 Rule 18

In addition to the letters above, the Zero-Width Non-Joiner (ZWNJ, U + 200C) functions as a morpheme boundary marker within single words in Badini Kurdish — a convention rooted in the foundational alphabet design of Bedirxan [15]. For example,  (we) contains a ZWNJ between  and  to prevent cursive joining while marking the clitic boundary. Legacy texts frequently encode this character as an apostrophe (U + 0027) or omit it entirely. Table 5 summarises the eight Kurdish-specific characters requiring normalisation, all of which are corrected by BDPT Stage 2.

These character-level issues cannot be resolved by tools designed for Arabic, Persian, or Sorani, as those dialects use different codepoints for the same visual glyphs. BDPT Stage 2 is specifically designed to detect and correct all eight codepoint errors shown in Table 5, applied in fixed rule order to ensure no rule conflicts.

### 4.2 Corpus collection

The Dasinya corpus was assembled from two institutional sources. The first is the Badirxanian Public Library, a government public library in Duhok, Kurdistan Region of Iraq, which provided 98 books and documents in legacy Microsoft Word format (.doc and .docx) spanning six book sub-genres: literary, historical, cultural, scientific, psychological, and educational. The second is the Semantic Web Lab (SWLab) at the University of Zakho, which provided 9 digitised documents: 4 books, which fall within the six book sub-genres listed above, and 5 non-book files spanning three non-book sub-genres — news, academic/linguistic, and broadcast. All 107 source files are in the Badini Kurdish dialect written in Perso-Arabic script. Files were received in their original format without preprocessing; legacy .doc files were converted to .docx using Microsoft Word via COM automation (pywin32) prior to pipeline processing.

The conversion from legacy .doc to .docx was performed by Microsoft Word driven through COM automation (the pywin32 library), saving each file in Word’s default Open XML format. Using Word’s own engine to convert its own legacy format maximises textual fidelity, but the output was not assumed to be lossless: two classes of artefact were anticipated and explicitly controlled for. First, conversion can carry over residual formatting and control characters — invisible Unicode characters, encoding artefacts, and structural debris such as page numbers and running headers — inherited from the original book layouts. Second, and more consequentially for a low-resource language, files authored with legacy Kurdish keyboard layouts and non-standard fonts can exhibit Perso-Arabic codepoint drift, in which a visually correct glyph is stored under an incorrect Unicode codepoint (for example, Arabic Yeh U + 064A in place of Kurdish Yeh U + 06CC).

No silent character losses affecting the Badini text were observed. Rather than treating the converted output as clean, every file was passed through the full validation and cleaning sequence, in which each artefact class is addressed at a dedicated stage: encoding and script validation with an audit log (Stage 1, Section 4.3); codepoint normalisation across 28 fixed-order rules (Stage 2, Section 4.4); line-level removal of non-linguistic structural content (Stage 3, Section 4.5); and risk-score-gated routing of files with residual invisible characters and encoding artefacts (Stage 4, Section 4.6). The conversion therefore surfaced codepoint irregularities for systematic correction rather than deleting content.

Particular care was taken to preserve the Zero-Width Non-Joiner (ZWNJ, U + 200C), which functions as a morpheme-boundary marker in Badini Kurdish and is precisely the character that an unguarded conversion or an aggressive cleaning step would be liable to strip. ZWNJ integrity was verified by comparing occurrence counts before and after processing: across the 107 admitted files, ZWNJ occurrences rose from 145,539 in the converted source to 646,829 in the processed corpus, an increase produced by the documented orthographic-normalisation rules rather than any loss. The ZWNJ is preserved across all 28 Stage 2 rules and is explicitly excluded from Stage 4 risk scoring for this reason.

### 4.3 Stage 1 — validation

**Fig. 3:**
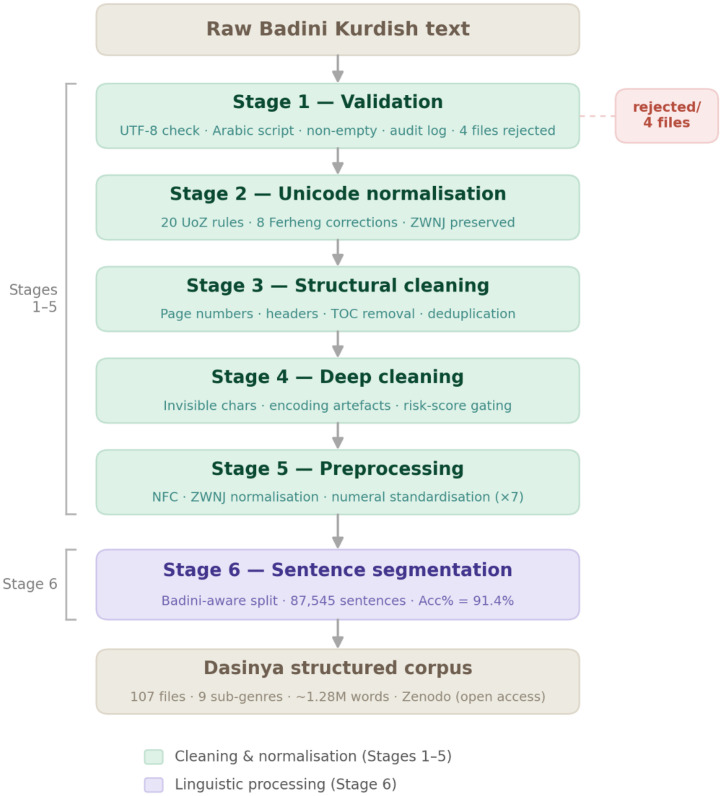
BDPT preprocessing pipeline architecture — six stages from raw Badini Kurdish text to structured corpus output. Teal: cleaning and normalisation (Stages 1–5). Purple: linguistic processing (Stage 6).

Every source file passes through Stage 1 before entering the pipeline. Three checks are applied: the file must be non-empty, must contain Perso-Arabic script text (Unicode block U + 0600–U + 06FF), and must be UTF-8 encoded. Any file that fails a check is placed in a rejected/ folder and excluded from all further stages; files that pass proceed to Stage 2. Every decision is written to an audit log (validation_log.csv). Of the 111 candidate files submitted, 107 were admitted and 4 were automatically excluded. emp.docx and Vala.docx were rejected because they carried no text content (too_little_text). The remaining two rejections were language-based: Ar-article.docx was excluded because its arabic_word_hits count (521) vastly outweighed its badini_word_hits (1), and farsi (news).docx was excluded because its persian_word_hits count (151) far exceeded its badini_word_hits (2). This automated gate prevents typographically similar but linguistically distinct material from contaminating the corpus without any human reviewer noticing. The full six-stage pipeline architecture is illustrated in Fig. 3.

### 4.4 Stage 2 — Unicode normalisation

Stage 2 applies Unicode normalisation rules specific to Badini Kurdish in two sequential sets. The first set comprises 20 character-mapping rules derived from the University of Zakho orthographic standard (UoZ rules), which correct codepoint errors introduced by legacy Kurdish keyboard layouts. The complete rule set is documented in Table 6. The second set comprises 8 orthographic corrections derived from the Badini spelling dictionary [16], documented in Table 7. Both sets are applied in fixed order — order is critical because some rules depend on the output of earlier ones (for example, Rule 10 maps Arabic Theh to a temporary ASCII marker ‘e’, which Rule 11 immediately maps to Kurdish P پ, preventing any false match on the letter ‘e’ in Arabic-script text). Rule 18, which maps the apostrophe character (U + 0027) to the Zero-Width Non-Joiner (ZWNJ, U + 200C), follows the morpheme boundary convention established for written Kurdish by Bedirxan [15], in which the apostrophe marks non-joining boundaries within a single word. The ZWNJ is explicitly preserved across all 28 rules, as it functions as a morpheme boundary marker within single words in Badini Kurdish [15].

**Table 6:** UoZ character-mapping rules applied in Stage 2 (source: Semantic Web Lab, University of Zakho). Rules are applied in the order shown; order is critical.

**Table 7:** Badini orthographic corrections applied in Stage 2 (source: Abdullah, 2021). Applied after the UoZ rules as a second normalisation pass.

The two-pass design — UoZ rules first, Ferheng corrections second — ensures that character-level codepoint errors are resolved before word-level spelling corrections are applied. All 28 rules are implemented in bdpt/stage2_unicode.py, which is included in the Zenodo repository.

### 4.5 Stage 3 — structural cleaning

Stage 3 removes non-linguistic content introduced by the legacy book format. Operations include removal of page numbers (isolated numerals on standalone lines), table of contents entries (title fragment followed by a page number), repeated headers and footers (identified by string matching across consecutive pages), and duplicated paragraph blocks. Structural cleaning operates at the line level; no characters within retained lines are modified, in accordance with the conservative cleaning principle applied throughout the pipeline.

### 4.6 Stage 4 — deep cleaning

Stage 4 removes invisible characters and encoding artefacts that survive Unicode normalisation. Lines that consist entirely of such noise — whole-line URLs, residual table rows, pure-punctuation lines, exercise checkboxes, dotted leaders, and Latin-script fragments left by legacy-font corruption — are discarded at the line level, while Arabic-script characters and the ZWNJ are never modified. Alongside this line-level cleaning, each file is also assigned a document-level risk score that gauges how much such noise was identified in it and gates its routing, without itself deleting any content. The score sums fixed ordinal weights over seven noise signals detected while processing the file — web addresses (weight 3), phonetic or linguistic notation (3), pipe-delimited table residue (2), pure-punctuation lines (2), checkbox exercise lines (2), garbled Latin–Perso-Arabic fragments (2), and dotted-leader lines (1) — for a maximum of 15. The weights are ordinal severity tiers rather than fitted coefficients: signals that most strongly indicate off-target material carry the highest weight, structural residue an intermediate weight, and cosmetic noise the lowest. Because the score is additive, a file is routed to manual review only when several independent noise signals co-occur. Files scoring at or below the threshold of 8 pass to the NLP-ready set; files scoring above 8 are routed for inspection. Across the 107-file corpus, scores ranged from 0 to 7 (mean 1.82), so the threshold sits one point above the observed maximum and every file passed automatically without manual review; the routing outcome is insensitive to the exact threshold within the range 8 to 15. The ZWNJ (U + 200C) is excluded from all scoring and removal logic.

### 4.7 Stage 5 — preprocessing

Stage 5 applies seven text preprocessing operations in sequence: (1) NFC Unicode normalisation to canonicalise composed character forms; (2) ZWNJ preservation check; (3) Arabic numeral unification, converting Eastern Arabic-Indic digits (٠–٩) to Western Arabic digits (0–9); (4) diacritic standardisation for Kurdish-specific vowel markers; (5) whitespace normalisation; (6) punctuation standardisation, unifying quotation mark and dash variants to canonical Kurdish forms; and (7) removal of URLs and non-linguistic tokens identified by pattern matching. All seven operations are applied per line in order; no operation modifies ZWNJ characters.

### 4.8 Stage 6 — sentence segmentation

**Fig. 4:**
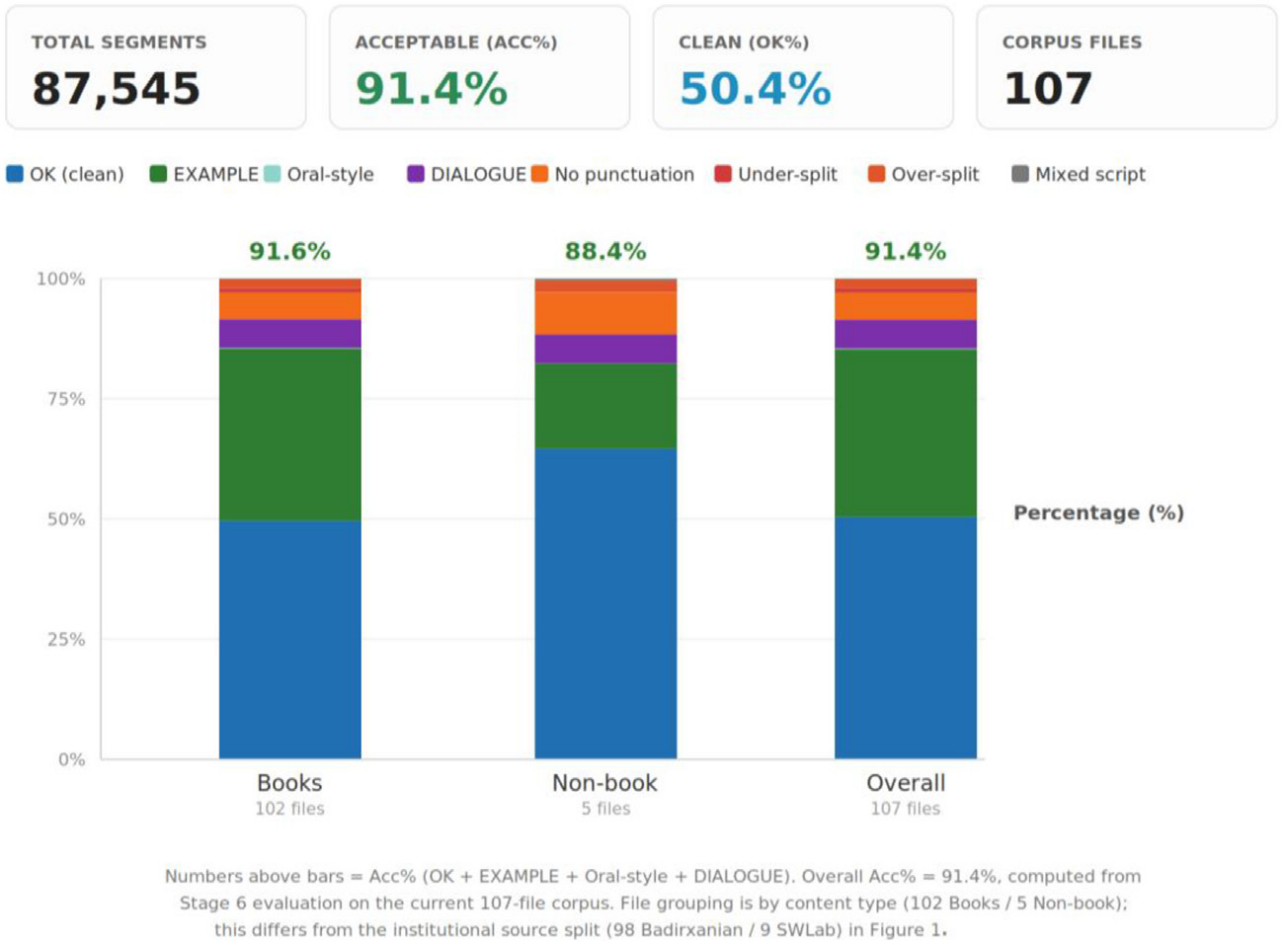
Stage 6 sentence segmentation evaluation results by genre. Each stacked bar shows the proportion of segment classes (OK, EXAMPLE, Oral-style, DIALOGUE, and the error classes NO_PUNCT, OVER_SPLIT, UNDER_SPLIT, MIXED_SCRIPT) per genre group; the value above each bar is Acc% (OK + EXAMPLE + Oral-style + DIALOGUE). Overall Acc% = 91.4% across 87,545 segments from 107 files.

The Stage 6 segmentation output (corpus/structured/) stores one .txt file per source document. Each file contains tagged segments using three markers: 〈T〉 (title-level segment), 〈P〉 (paragraph-level segment), and 〈S〉 (sentence-level segment). The evaluation results per genre group are summarised in Table 8, with genre-level proportions visualised in Fig. 4.

**Table 8:** Stage 6 sentence segmentation results per genre group.

Genre group	Segments	Share	OK%	Acc%
Books (all)	83,320	95.2%	49.6%	91.6%
Non-book (all)	4225	4.8%	64.6%	88.4%
ALL FILES	87,545	100%	50.4%	91.4%

Stage 6 performs Badini-aware sentence segmentation using a rule-based approach configured via a SEGMENTATION_CONFIG object specifying boundary markers (Kurdish full stop, Arabic full stop, exclamation mark, question mark), minimum and maximum segment length thresholds, and genre-specific connector rules that prevent over-splitting at coordinating conjunctions. Sentence boundaries are identified following the formal definition of a Kurdish sentence as a collection of speech elements arranged according to language rules in order to express one or more ideas at a given moment [17]. Each output segment is tagged with one of three markers: 〈T〉 for title-level segments, 〈P〉 for paragraph-level segments, and 〈S〉 for sentence-level segments.

**Fig. 5:**
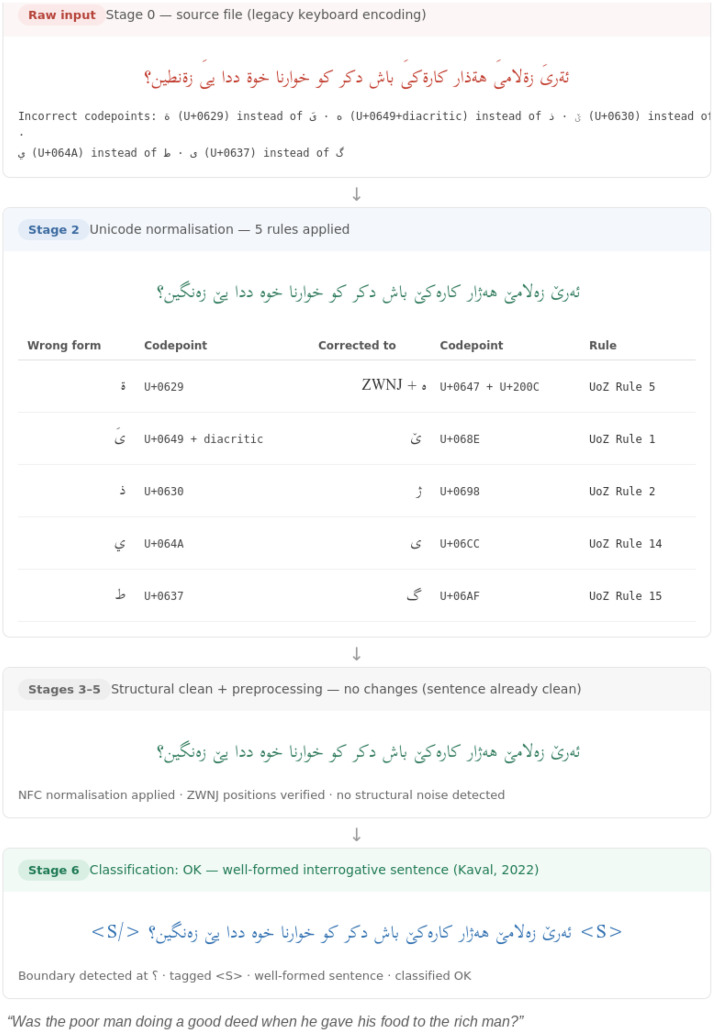
Example sentence transformation through the BDPT pipeline. Five Unicode errors corrected by Stage 2 [15]. Stage 6 classifies as OK.

All 107 corpus files (87,545 segments in total) were evaluated under a two-metric framework. OK% captures the share of fully well-formed, punctuated sentences. Acc% is broader: it counts OK segments together with EXAMPLE segments (word lists, vocabulary exercises, and diary entries that the pipeline correctly identifies as non-sentence content), Oral-style segments (intentionally long oral-register broadcast and social-media units), and DIALOGUE segments (direct speech). Over the full corpus, OK% reached 50.4% and Acc% reached 91.4%, a result validated by a Kurdish language specialist. Genre-specific thresholds were necessary because the sub-genres of Dasinya follow substantially different textual conventions that make a single cut-off unsuitable. Educational books are built around word lists and exercises that are valid corpus content but are not sentences; applying a sentence-length threshold to them would misclassify acceptable material as errors. Historical writing favours long, clause-chained sentences by convention, so segments that exceed a standard maximum length represent authentic prose, not under-splits. Academic and linguistic texts include IPA notation and technical symbols that are correctly labelled MIXED_SCRIPT rather than treated as errors. Imposing one uniform threshold across all these genres would either penalise the pipeline for handling genre conventions correctly, or introduce distortions in the source material. The genre-aware approach taken here is consistent with established practice in sentence boundary detection for heterogeneous corpora [18]. A concrete example of the full transformation from raw legacy text to a Stage 6 classified sentence is shown in Fig. 5.

## Limitations

Dasinya covers only the Badini Kurdish dialect in Perso-Arabic script; the Latin-script Kurmanji variety and all other Kurdish dialects fall outside its scope. The corpus is drawn from two institutional repositories — the Badirxanian Public Library and the Semantic Web Lab at the University of Zakho — and does not capture every register of written Badini Kurdish: spoken transcripts, informal handwritten texts, and specialised domains such as legal or medical writing are absent.

All source files arrived in legacy .doc or .docx format, which carried encoding artefacts requiring correction at the pipeline level. Even after six stages of processing, 8.6% of segments remained non-acceptable (classified as NO_PUNCT, OVER_SPLIT, UNDER_SPLIT, or MIXED_SCRIPT). This residual rate traces primarily to irregular punctuation in the original books rather than to weaknesses in the pipeline itself.

Reproducing the corpus from the original .doc sources additionally requires Microsoft Word on Windows for the initial format conversion, although the released .docx inputs and all downstream pipeline stages run on any platform with Python.

Tokenisation (Stage 7), stemming (Stage 8), stopword removal (Stage 9), and part-of-speech tagging are planned as future work.

## Ethics Statement

Both authors have read and comply with the ethical requirements for publication in Data in Brief. No human participants, animal experiments, or social media data collection were involved in this research. Every source text in the Dasinya corpus was obtained from two institutional repositories — the Badirxanian Public Library in Duhok and the Semantic Web Lab at the University of Zakho — and consists entirely of published books or institutional documents. No personal data of any kind were collected or processed.

## Funding

This research did not receive any specific grant from funding agencies in the public, commercial, or not-for-profit sectors.

## Ethics

This work did not involve human participants, animal experiments, or the collection of any personal data. All source texts in the Dasinya corpus were obtained from institutional repositories — the Badirxanian Public Library, Duhok, and the Semantic Web Lab, University of Zakho — and consist entirely of published books or institutional documents.

## Originality and Consent to Submit

The authors confirm that this manuscript is original, has not been published previously, and is not under consideration for publication elsewhere. All authors have read and approved the revised manuscript and agree to its resubmission to Data in Brief.

## Data Availability

The Dasinya corpus and the BDPT preprocessing pipeline are openly available in the Zenodo repository: Dasinya — Badini Kurdish Corpus and BDPT Pipeline, https://doi.org/10.5281/zenodo.20729060 [19].

## CRediT authorship contribution statement

**Vaman A. Saeed:** Conceptualization, Data curation, Methodology, Software, Formal analysis, Investigation, Writing – original draft, Writing – review & editing. **Karwan Jacksi:** Supervision, Conceptualization, Writing – review & editing, Validation.
